# Application of the Behaviour Change Wheel to Optimise Infant Feeding in Bangladeshi and Pakistani Communities in the UK: Co‐Development of the Learning About Infant Feeding Together (LIFT) Intervention

**DOI:** 10.1111/mcn.70019

**Published:** 2025-04-24

**Authors:** Kayleigh Kwah, Naomi Bartle, Maxine Sharps, Kubra Choudhry, Jackie Blissett, Katherine Brown

**Affiliations:** ^1^ Public Health and Applied Behaviour Change Laboratory University of Hertfordshire Hatfield UK; ^2^ Department of Health and Life Sciences School of Applied Social Sciences, De Montfort University Leicester UK; ^3^ School of Social Sciences and Humanities Coventry University Coventry UK; ^4^ School of Life and Health Sciences, Aston University Birmingham UK

**Keywords:** behavioural research, breast feeding, community‐based participatory research, culture, feeding behaviour, human, milk, psychosocial intervention

## Abstract

Breastfeeding rates in the UK are amongst the lowest in the world, largely driven by individual‐ and social‐level barriers. Evidence has also highlighted that cultural factors can play an important part, such as for the UK South Asian community. Although aggregated breastfeeding data indicates that initiation is high amongst the UK South Asian population, sub‐group data shows that this is substantially lower amongst people of Pakistani and Bangladeshi ethnicity. As such, culturally tailored interventions are called for. This research aimed to systematically develop an evidence‐based culturally tailored intervention to support the optimisation of infant feeding in these communities. The ‘Learning about Infant Feeding Together’ (LIFT) intervention was co‐developed by researchers, six community peer group champions, and a 3rd sector organisation supporting UK South Asian women. Development was guided by the REPLACE approach (a framework for the development of culturally specific community‐based interventions) and the Behaviour Change Wheel (a framework for describing, designing and evaluating behaviour change strategies). It involved three co‐development intervention workshops as part of a rigorous systematic intervention development approach. A culturally tailored intervention incorporating nine behaviour change techniques was produced. The intervention aims to increase breastfeeding by targeting six infant feeding behaviours identified as important, changeable and pertinent to the communities involved. The final intervention includes posters, leaflets, and an animation. The transparent reporting of intervention content and the approach taken to development will support the growth of evidence‐based practice in the field of infant feeding.

## Summary

- There is evidence of culturally driven infant feeding behaviours and barriers to breastfeeding amongst UK Pakistani and Bangladeshi communities.
- Formative research and co‐development with the target population aids in the development of culturally appropriate and sensitive interventions.
- The LIFT intervention was rigorously developed following theory‐based behaviour change frameworks (REPLACE approach and the Behaviour Change Wheel) but careful consideration and planning of co‐development work with community members is required.

## Graphical abstract

This research provides a demonstration of successfully co‐developing a culturally appropriate intervention for infant feeding, following principles of theory‐based frameworks, guided by high quality formative research to elucidate the specific socio‐cultural beliefs impacting infant feeding in the UK Bangladeshi and Pakistani population. Intervention materials were designed to complement face‐to‐face infant feeding support.

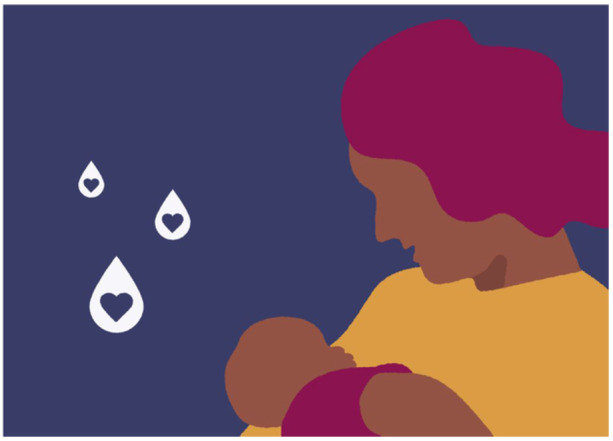

**Abbreviations**
APEASE: affordability, practicability, effectiveness/cost‐effectiveness, acceptability, side‐effects/safety and equity
BCW: behaviour change wheel
COM‐B: capability, opportunity, motivation and behaviour
FWT: Foleshill Womens Training
LIFT: learning about infant feeding together
MRC: Medical Research Council
REPLACE: Researching Female Genital Mutilation Intervention Programmes linked to African Communities in the EU

## 1 Introduction

An established body of evidence indisputably demonstrates that breastfeeding has positive outcomes for both mother and infant (Horta et al. 2007; Renfrew et al. 2012; Victora et al. 2016). The World Health Organisation recommends that an infant receives breast milk exclusively for the first 6 months of their life and continues to receive it up until at least 2 years of age alongside the introduction of complementary foods (World Health Organization 2001). In the UK, few infants receive this (Office for Health Improvement and Disparities 2021), with mothers citing significant barriers rooted in both individual, social and organisational level factors (National Guidelines Alliance [UK] 2021). The Infant Feeding Survey (IFS), last conducted in 2010, reported on the UK's overall breastfeeding rate as well as on that for sub‐populations defined by ethnicity and other socio‐demographic characteristics (McAndrew et al. 2012). Aggregated data report that the South Asian population in the UK had relatively high breastfeeding initiation rates (96%), implying a low need for breastfeeding support. However, a re‐analysis highlighted that high initiation rates in Indian communities masked substantially lower rates in Pakistani and Bangladeshi communities. Whilst those of Indian ethnicity had initiation rates equivalent to the South Asian group percentage (97%), those of Pakistani (79%) and Bangladeshi (85%) ethnicity had substantially lower rates, closer to that of the UK's average (87%), with similar trends at 6 weeks (Choudhry 2018; McAndrew et al. 2012). The IFS was discontinued in 2010 with national data subsequently reported by region but without reporting on data by ethnicity. These statistics demonstrate little change in the UK's overall breastfeeding initiation and continuation rates (Office for Health Improvement and Disparities 2021) but whether differences by ethnicity persist is unknown. The IFS has recently been reinstated but to date no data has been released to confirm this.

As well as ‘universal’ breastfeeding barriers, evidence has outlined additional cultural factors that can influence breastfeeding behaviour amongst sub‐populations defined by ethnicity. For the South Asian community these include; delaying breast milk or discarding colostrum (Choudhry 2018; Cook et al. 2021); pre‐lacteal feeding (Choudhry 2018); complementary feeding before the age of 6 months, often as a result of baby's weight (Lakhanpaul et al. 2020); ‘pardah’ (modesty) (Choudhry 2018); and conflicting pressures from older generation family members (Choudhry and Wallace 2012). Such cultural norms and practices can have a negative impact on establishing breastfeeding and can result in the early introduction of formula milk. Beliefs, social norms and cultural practices that impact breastfeeding differ by ethnicity but also by community (Cook et al. 2021; Ingram et al. 2003; Kwah et al. 2025). This emphasises the importance of recognising, understanding and addressing specific cultural differences and the wider impact they can have, when designing intervention efforts (Lakhanpaul et al. 2020; Noor and Rousham 2008). Despite efforts to improve the cultural competency (knowledge, understanding and respect for differences across cultures) of UK maternity and breastfeeding services, research demonstrates further work is needed before this is achieved to a high and consistent standard (Hassan et al. 2020; McFadden et al. 2013; Wilde 2023). Recommendations to embed education about cultural awareness, sensitivity and competence for professionals providing support with breastfeeding (Gutierrez 2022), indicates the need for tailored resources for health professionals to support parents.

To date, only a small number of interventions have attempted to impact infant feeding behaviours in the first 6 months of life in the South Asian population in the UK. Two noteworthy interventions are those developed by Ingram and colleagues (Ingram et al. 2003; Ingram and Johnson 2004) who utilised formative research and co‐designed them with the target population. These were delivered to expectant South Asian mother‐grandmother or mother‐partner pairs. The authors concluded that both interventions, which were similar in content and delivery, appeared to have a positive impact on behaviours, such as discarding colostrum, and giving water or formula milk (Ingram et al. 2003), and were reported as acceptable, useful and enjoyable by users (Ingram and Johnson 2004). Despite this encouraging research, limited conclusions can be drawn about the active components of these interventions due to the sparse reporting of content. Guidance outlined by the Medical Research Council (MRC), a UK funding organisation that supports research to improve human health, states that the development of complex behaviour change interventions should be guided by evidenced‐based methodology, and be explicitly described, to identify mechanism of action and to support replication (Skivington et al. 2021) Infant feeding interventions described in the literature typically provide little or no information on the process of development, or on whether/how they have been informed by theory or evidence. Furthermore, sparsely reported intervention descriptions means that specific behaviour change content cannot be identified or evaluated. This means that when evaluating infant feeding interventions, we cannot be clear as to what works or why. The Behaviour Change Wheel (BCW) (Michie et al. 2011) offers a practical step‐by‐step evidence‐based framework for designing behaviour change interventions, underpinned by the COM‐B model (a model based on 19 behaviour change models which broadly categorises behavioural determinants into (a) capability, (b) opportunity and (c) motivation). The BCW guides the identification of behaviours to target, what needs to change (capability, opportunity and motivation) to achieve the target behaviour, and what potential intervention functions and behaviour change techniques (BCTs) could be used to design intervention content. Utilising and reporting intervention development in line with frameworks such as COM‐B ensures that we can begin to build a science of what works to address infant feeding for specific population groups and contexts.

The co‐development of interventions with the target population is also recommended by the MRC (Skivington et al. 2021). This ensures that interventions are appropriate given the specific and unique needs of the intended end user. The co‐development process ensures that the voices of individuals and communities are heard in efforts aiming to directly improve health and reduce health inequalities. Including these voices is not only an avenue to democratise research (Beresford 2013) but has been shown to positively impact on those involved in the co‐design process and on the acceptability and applicability of subsequent research materials (Slattery et al. 2020). Despite this, co‐designed research with diverse ethnic minority communities is rare.

This paper outlines the systematic approach taken to co‐develop the ‘Learning about Infant Feeding Together’ (LIFT) intervention. LIFT is an theory based culturally tailored infant feeding intervention aimed at the Pakistani and Bangladeshi communities residing in the UK. Intervention development was guided by the REPLACE approach (a framework for the development of community‐based interventions which recommends the application of both individual and community‐level behaviour change theories to support and inform the co‐production of interventions (Barrett and Brown 2015) supplemented with the Behaviour Change Wheel guidance (Michie et al. 2014). In line with best practice guidance (Skivington et al. 2021) this paper provides a detailed description of the process of development and of intervention content.

## 2 Methods

### 2.1 The LIFT Project Team

The LIFT project team comprised of a team of Health and Developmental Psychology researchers (NB, KB, JB, KK, KC, MS), representatives of Foleshill Women's Training (FWT; a community organisation working with women from South Asian communities to promote health and wellbeing), and six community peer group champions. The community peer group champions were identified and recruited during the period of formative research that preceded the intervention development (see Kwah et al. 2025). Advertisements were placed within FWT's centre and distributed during a family event and community workshops run during the community engagement element of the formative research. The community peer group champions were mothers from the community who had some involvement with FWT, had attended the community workshops as part of the formative research, but had no prior experience of academic research. The role of the community peer group champions was to voice the beliefs, needs and considerations of their community during the process of co‐development and co‐develop an acceptable and culturally specific infant feeding intervention.

### 2.2 Design

The development of the LIFT intervention was guided by the REPLACE approach (Barrett and Brown 2015), a framework for the development of culturally specific interventions. The approach comprises 5 elements; (1) community engagement; (2) Understanding social norms; (3) community readiness to change; (4) intervention development; (5) evaluation. The first three elements of this approach (referred to as ‘formative research’) are reported elsewhere (Kwah et al. 2025). Element four, the focus of this paper, utilises evidence gathered during these earlier phases to develop the intervention content. Alongside the REPLACE manual's guidance on intervention development more detailed guidance of the Behaviour Change Wheel (Michie et al. 2014) was followed. Fidelity to REPLACE guidance on conducting element four was maintained, for example, the continued focus on community involvement.

**Figure 1:**
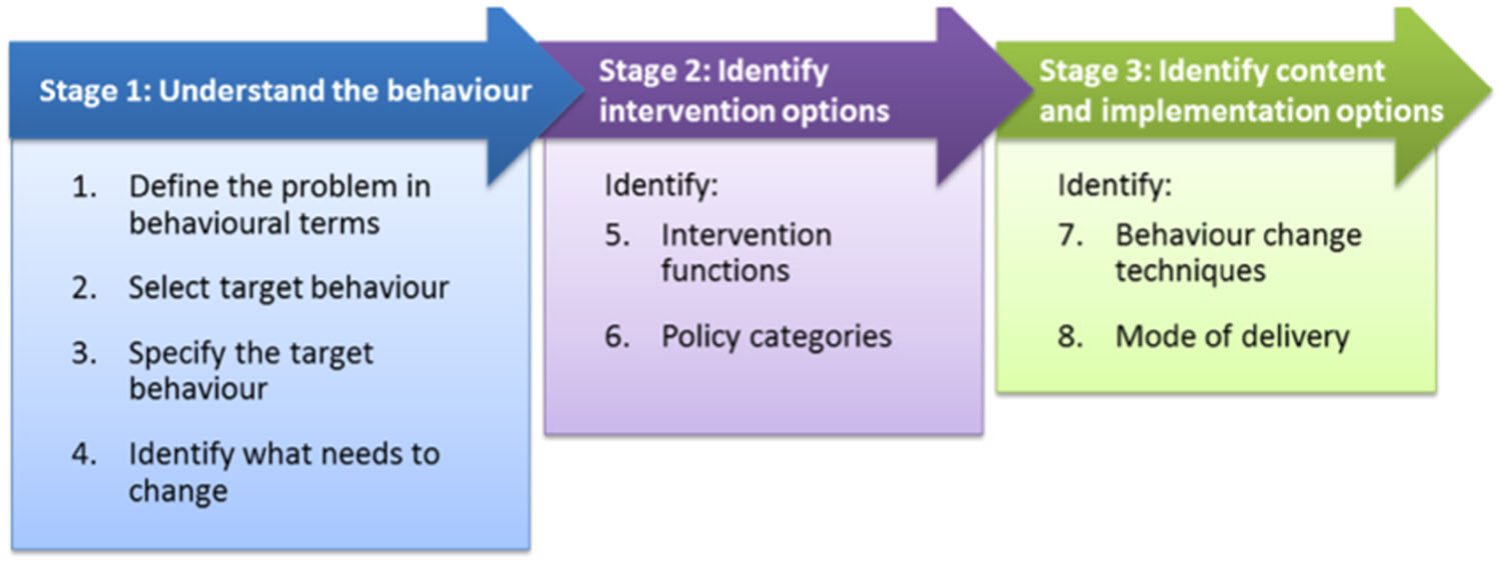
Behaviour change wheel intervention design process.

The steps outlined below are aligned to those described in the Behaviour Change Wheel guidance (Michie et al. 2014); see Figure 1. Information on additional considerations or activity in line with the RELACE approach are also provided where relevant. Across these steps, three intervention co‐development workshops were held. These are described in detail in the context of the steps taken. The Behaviour Change Wheel provides a framework for the development of evidence‐based interventions, following eight steps conducted consecutively and iteratively (see Figure 1).

All workshops were attended by the community peer group champions, at least two researchers, and two representatives of FWT. Each workshop was structured so that any activities were preceded with relevant content and information giving. To ensure all members of the group were able to share their thoughts, a range of physical materials were provided (e.g., post it notes and flip chart paper and pens), but in most cases, the scribing was completed by a member of the LIFT team at the request of attendees. This allowed the community peer group champions to communicate their thoughts verbally, which was usually their preferred method for contribution.

### 2.3 Intervention Development

#### 2.3.1 Step 1: Define the Problem in Behavioural Terms

The purpose of this step is to identify the behaviour(s) that needs to be changed, and then to specify the location in which it occurs and the individual, group or population concerned. First, existing research (including the formative research conducted as part of elements 1–3 of the REPLACE approach), and national‐level infant feeding statistics and guidance, were collated. This information was then presented at the first of the three intervention development workshops. Discussion followed to begin the process of specifying the target behaviour(s), location and population.

#### 2.3.2 Step 2: Select the Target Behaviour

The purpose of this step is to identify interdependent behaviours and then to prioritise these for intervention; this aligns with the REPLACE activity of ‘identifying target actions’. This activity was also performed at the first workshop. To assist in this exercise, the research team presented findings from element 3 ‘community readiness to change’ of the REPLACE approach conducted during the formative research (reported elsewhere; see Kwah et al. 2025) whereby key infant feeding beliefs and practices of Pakistani and Bangladeshi communities had been grouped and rated in terms of the perceived readiness of the community to change. Splitting into small groups (each including community peer group champions, a researcher, and a representative from FWT) work was then undertaken to identify interdependent behaviours (i.e., behaviours that responded to culturally driven infant feeding practices identified as having a negative impact on the initiation and/or duration of breastfeeding), and then to discuss and agree which should be prioritised for intervention. Printed materials to support the prioritisation activity were taken along to the workshop. These directed the groups to discuss the likelihood that each behaviour could be changed and whether changing the behaviour might have any unintended positive or negative consequences on breastfeeding (‘spillover effects’). Groups were also asked to use the readiness to change ratings to inform their decision making. Readiness to change was classified, as low, medium, or high, using a 9‐point scale as a guide (see Supporting Information ‘Long List of possible intervention target behaviours generated during the first intervention development workshop’).

#### 2.3.3 Step 3: Specify the Behaviour

The purpose of Step 3 was to add further precision to the behavioural specification. Again, as part of Workshop 1, small group discussions were held for each of the identified behaviours to answer the following questions; *what* do they need to do differently to achieve the desired change?; *when* do they need to do it?; *where* do they need to do it?; *how often* do they need to do it?; and *with whom* do they need to do it? In line with the REPLACE approach, specific attention was given to identification of culturally‐specific norms and enforcing mechanisms that may influence each of these behaviours and what, if any, additional considerations ought to be made in light of this.

#### 2.3.4 Step 4: Identify What Needs to Change

**Figure 2:**
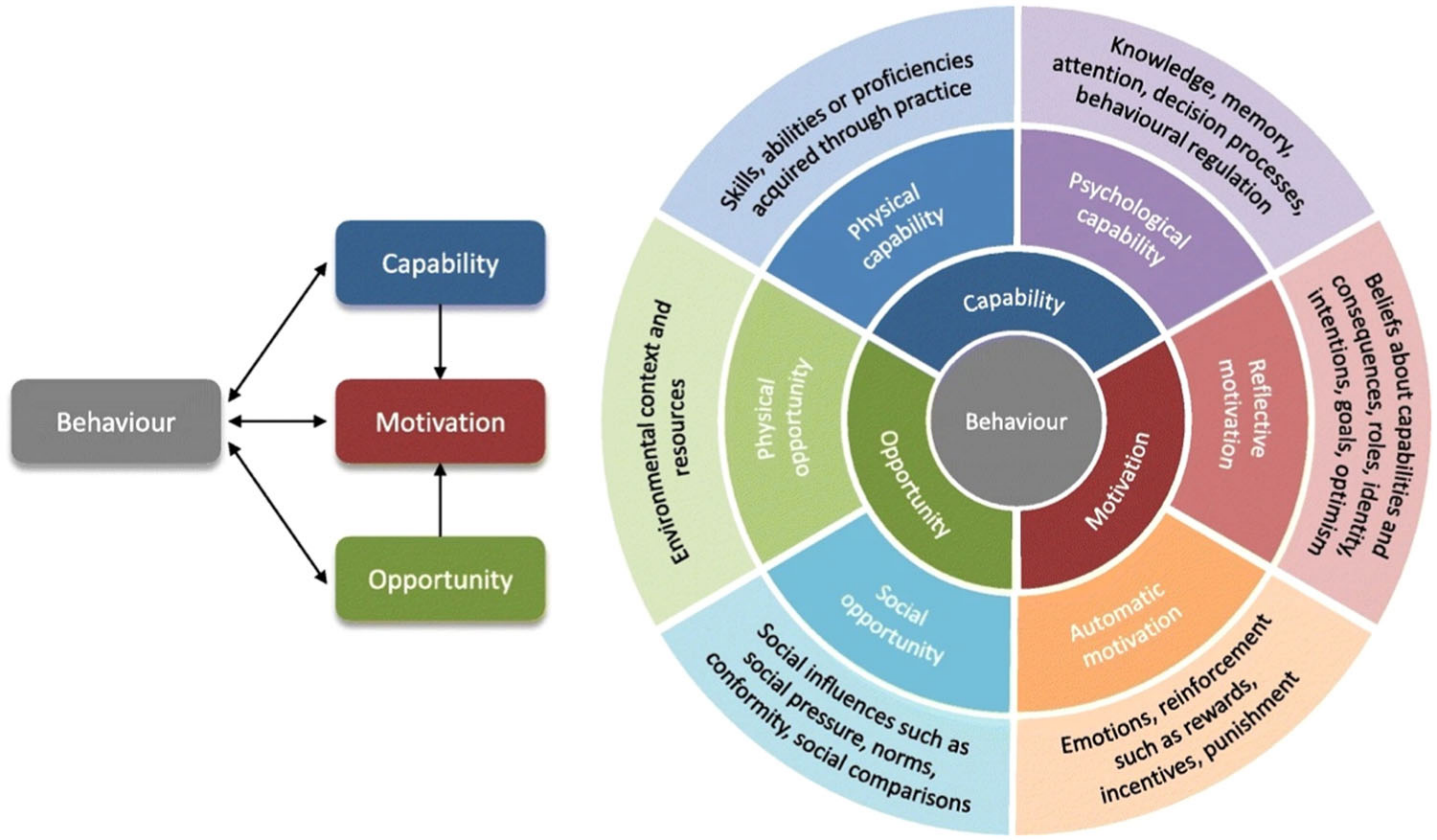
The COM‐B model.

The purpose of Step 4 is to identify what needs to change at the level of the person or environment to achieve desired change in the target behaviours. In line with the Behaviour Change Wheel, the COM‐B model (see Figure 2) was used to this end. The COM‐B posits that for any *Behaviour* to occur, there must be, ‘*Capability*’ to do it, ‘*Opportunity*’ for it to occur, and sufficiently strong ‘*Motivation*’ (Michie et al. 2014). Identification of what needs to change was performed in partnership with the community peer group champions via Workshop 2. This workshop began with a presentation during which a plain English description of the COM‐B model was provided and the primary and target interdependent behaviours identified at the last workshop were revisited. Some scenario‐based activities to aid understanding of the COM‐B model and its application were conducted. This was done by providing an example of a behaviour (e.g., not giving tastes of food and drinks before 6 months) and context (grandparent visiting and offering tastes of food or drink, other than breast or infant formula milk, to infant) and asking the community peer group champions to think about the barriers and facilitators (what would be helpful and what would be challenging in this situation). These were recorded on worksheets and prompt cards were given out to facilitate thinking (e.g. family; culture; health; information). A COM‐B analysis at an appropriate level for the community peer groups champions' knowledge and the time available was completed. Researchers made some refinements to the COM‐B analysis, utilising the more granular detail of the Theoretical Domains Framework (TDF) (illustrated in the outer circle of Figure 2) to understand the behaviour, following the workshop using the knowledge gained from the intervention development workshop alongside knowledge gained during the formative research (Kwah et al. 2025).

#### 2.3.5 Steps 5–8: Intervention Functions; Policy Categories; BCTs; Mode of Delivery

Using the COM‐B analysis from Step 4, and guided by the Behaviour Change Wheel approach, the research team completed Steps 5, 6 and 7 to identify which interventions functions, policy categories and BCTs should be considered. During the third and final workshop, the research team then presented a set of key intervention messages, along with potential intervention functions and BCTs, to the rest of the LIFT project team. Information about the variety of delivery methods using examples of existing intervention materials were also introduced. When deciding on intervention ideas, community peer group champions were asked to consider which were the most appropriate for their community. This was guided by researcher prompted conversation and drew on the APEASE criteria defined as Affordability, Practicability, Effectiveness/Cost‐effectiveness, Acceptability, Side‐effects/Safety and Equity (Michie et al. 2014).

### 2.4 Ethical Statement

Ethical approval was received from Coventry University ethics committee.

## 3 Results

### 3.1 Step 1: Define the Problem in Behavioural Terms

The collated evidence identified that: breastfeeding rates within Pakistani and Bangladeshi communities are lower than that of other South Asian groups living in the UK (Choudhry 2018), that cultural beliefs and practices held by these communities (summarised in Table 1) influence breastfeeding, and that these beliefs and practices are not fully understood. There is variability in how these are addressed within health services (Hassan et al. 2020; McFadden et al. 2013; Wilde 2023) and a distinct lack of theory driven co‐developed culturally tailored interventions. This provided a sound rationale for the development of an intervention and also a good understanding of the problem amongst the LIFT project team who then went on to agree the following behavioural specification:

What behaviour?: increasing the initiation and duration of breastfeeding.

Where does the behaviour occur?: non‐specific (behaviour could occur in any location).

Who is involved in performing the behaviour?: Pakistani and Bangladeshi mothers.

**Table 1:** Intervention target behaviours mapped onto the key cultural beliefs about infant feeding.

Key cultural belief/practice	Selected interdependent behaviour(s)
**Family relationships and the influence on infant feeding decisions:** The older members of the family unit have a significant influence on the decision about how the baby will be fed and often the mother‐in‐law requests that formula is used. Fathers do not have a role in infant feeding decisions/behaviours and do not tend to get involved. The baby's mother often has household/daughter‐in‐law duties to attend to before the baby and that makes it difficult to breastfeed/easier to formula feed. ‘Pardah’ or the need for modesty, makes bottle feeding easier and breastfeeding difficult in public.	Parents to talk to family members about breastfeeding to establish breastfeeding support
[Bangladeshi community] The first breast milk (colostrum), is perceived to be old and ‘dirty’ milk that should be discarded before feeding the baby.	Mothers to feed their baby colostrum (first breast milk)
‘Ghutti’, the practice of family elders giving baby either honey or dates (chewed up) before their first feed, passes on important qualities from grandparent to baby, and is an important cultural and religious practice.	Parents to avoid giving their baby honey before the age of 1 year
Babies offered liquid/food before 6 months of age (e.g., water mixed with dill seed) to help with their health (e.g., cleanse the baby's digestive system).	Parents to avoid giving their baby any taste of food or drink, aside from breast or infant formula milk, until they are at least 6 months old, and to also decline offers of this from other family members
Some mother's milk is not good enough quality and results in a smaller, less nourished baby, who needs to be given formula milk (to thrive). Formula milk results in bigger babies, bigger is viewed as better. ‘Pardah’ or the need for modesty, makes bottle feeding easier and breastfeeding difficult in public.	Parents to avoid or delay the use of formula milk in place of breast milk

### 3.2 Step 2: Select the Target Behaviour

The full list of behaviours identified as interdependent with breastfeeding are presented in Supporting Information ‘Long List of possible intervention target behaviours generated during the first intervention development workshop’. The sub‐group of these behaviours selected for intervention are presented above (Table 1). These are mapped against the key cultural beliefs and practices identified from prior formative research (Kwah et al. 2025). Whilst the intention was to select the target interdependent behaviours through a process of reviewing the ‘long list’ of identified behaviours and discussing/agreeing their relative importance, changeability, and potential for spill‐over, in practice this proved to be challenging because some of the concepts were not fully understood, and the peer group champions were more confident talking in more general terms. Rather than systematically reviewing and rating these elements, the small groups instead talked in more general terms about the behaviours. There was consideration of which were most important and of the ratings of community readiness to change generated through the prior formative research. For example, discussions were held about the ‘low’ rating that had been assigned for community readiness to address the practice of pre‐lacteal feeds. One of the identified interdependent behaviours relating to this practice was avoiding giving honey to babies under 1 year. It was discussed and agreed that this specific behaviour was important (given the risk of bacterial infection that can cause serious illness in infants) and that intervention on this specific behaviour may be acceptable to the community. It was therefore agreed that this would be included as an acceptable step related to the behvaiour ofof pre‐lacteal feeds.

### 3.3 Step 3: Specify the Behaviour

Table 2 presents the detailed specification of the target behaviours, with reference to cultural sensitivities where appropriate, as generated by the LIFT project team in Workshop 1.

**Table 2:** Specifying the target behaviours.

Behaviour specification						
Target behaviours	*Who* needs to perform the behaviour?	*What* does the person need to do differently to achieve the desired behaviour?	*When do* they need to do it?	*Where* do they need to do it?	*How often* do they need to do it?	*With whom* do they need to do it?
**Parents to talk to family members about breastfeeding to establish breastfeeding support**	Expectant and new parents	Parents talk to other family members who have a role in making decisions about infant feeding to discuss their feeding intentions and ways they can be supported	Before the baby is born (antenatally) and on‐going after the baby is born	Wherever feels comfortable, this might be in the family home	As required, before the baby is born, and whenever the topic of infant feeding arises before and after the baby is born	With the baby's other parent and family members who they feel have a role in decision and support around the way their baby is fed
	**REPLACE cultural considerations**					
	Cultural regard for elders is important which can make it hard for expectant or new parents to challenge their parents or parents‐in‐laws, when their beliefs about infant feeding may differ. Daughters‐in‐law may need to be particularly respectful of their mother‐in‐law and expectations on them in the family home—especially where there are intergenerational households					
**Mothers to feed their baby colostrum (first breast milk)**	Mothers of a newborn baby	Mothers of a new baby to breastfeed the first milk that they produce (colostrum)	Straight after the baby has been born and the following 2–3 days	Wherever the baby has been born, this could be in a medical setting or in their home	Every time the baby is feed	Alone; with support from other parent or care giver; with support from health professionals
	**REPLACE cultural considerations**					
	In the Bangladeshi community, there may be a cultural belief that colostrum is ‘dirty’ or ‘old’ milk that should not be fed to infants. This belief is sometimes passed down from older generations but is not always adopted by the younger generation.					
**Parents to avoid giving their baby honey before the age of 1 year**	Parents or other carers of a baby under 1 year	Parents to not give their baby honey and to decline if anyone else offers to give the baby honey before the age of 1	In the first few days of the baby's life when this might be offered; Until the baby is 1 years old	Wherever the baby is being offered honey, this might be a family home	For the first year of the baby's life and at any occurrence the baby is offered any amount of honey	The other parents or carers of the baby
	**REPLACE cultural considerations**					
	The traditional practice of giving pre‐lacteal feeds, such as honey, are believed to pass good qualities from elders to infant and are not considered harmful to the infant. Pre‐lacteal feeds may also be seen as a religious teaching.					
**Parents to avoid giving their baby any taste of food or drink, aside from breast or infant formula milk, until they are at least 6 months old, and to also decline offers of this from other family members**	Parents or other carers of a baby under 6 months old	Chose not to give baby any tastes of food or drink other than breast or infant formula milk and say no to family or community members if they offer to during the first 6 months of baby's life Parents need to feel confident to do this and feel knowledgeable about their decision	During the first 6 months of the baby's life and at every occurrence the baby is offered anything other than breast or infant formula milk	Wherever the offer is being made. This might be in their home, in their family's home, at a community venue or at hospital	At every occurrence when the baby is offered anything other than breast or infant formula milk during the first 6 months if the baby's life	The other parents or carers of the baby; other family members; community members
	**REPLACE cultural considerations**					
	Offering ‘tastes’ is not always viewed as offering food and is a cultural norm. Particularly if an infant is unwell, they may be given for example water with aniseed. Tastes are believed to have benefits to baby's health.					
**Parents to avoid or delay the use of formula milk in place of breast milk**	Parents or other carers of a baby under 6 months old	Decline offers of infant formula milk feeding from other family members if baby is exclusively breastfed	Each time a family member offers to give their baby formula milk	Wherever the offer of formula milk is being offered	At every occurrence when the baby is offered infant formula milk in place of breast milk	With support from other parents and care givers; with health professional support
	**REPLACE cultural considerations**					
	Older family members have a significant role in decisions about infant feeding, especially when living in extended families. Older family members are sometimes the source of comments about baby being ‘weak’ or milk not being good enough. There is a belief that formula makes babies big and strong and that breastfed babies are small. Expectations around mothers’ behaviour e.g. cannot breastfeed baby front of family/in public.					

### 3.4 Step 4: Identify What Needs to Change

A COM‐B analysis was conducted on each of the target behaviours and can be found in the supporting information (Supporting Information: Identifying what needs to change using the COM‐B model). One illustrative example is presented below in Table 3. This process was led more heavily by the researchers, as conducting the analysis in a truly collaborative way with the community peer group champions was unachievable. Informed by data gathered from the formative research and discussions and data gathered from the intervention workshops, in most cases the behavioural diagnosis revealed that all three components of the COM‐B model represented barriers to the behaviours.

**Table 3:** Identifying what needs to change (COM‐B analysis).

Target behaviour: Parents to talk to family members about breastfeeding to establish breastfeeding support		
COM‐B component	Evidence related to component & if relevant what needs to happen for the target behaviour to occur?	Is there a need for change?
Physical capability	There is no evidence that having the verbal and language skills to hold a conversation with family members is a barrier to establishing support to breastfeeding, as in most cases there are no communication or language barriers.	No
Psychological capability	Being able to explain their feeding choices and the knowledge base used to make this decision, to other family members, respectfully in the relationship they hold, was highlighted as a barrier to establishing breastfeeding support. Being knowledgeable about the benefits of breastfeeding and confident about the signs of healthy infant growth is faciliatory to these conversations. Furthermore, being able to express thoughts and feeling about infant feeding decisions, and any support needed, to elder family members is a facilitator to establishing breastfeeding support.	Yes
Physical opportunity	No evidence of physical opportunity being a barrier to establishing breastfeeding support, as parents are able to be physically present in a space with older family members.	No
Social opportunity	Mother‐in‐laws have a significant role in infant feeding decisions, especially when living in extended families and some have negative thoughts/feelings about breastfeeding, and this can act as a barrier to starting conversations. Parents need to feel like they can ask family members for support. Family members need to be open to having conversations about infant feeding benefits, decisions and the support needed, and open to hearing new knowledge‐based perspectives that might not align to their own.	Yes
Automatic motivation	Parents don't want to cause offence or be disrespectful of their older family members beliefs. Parents need to be empowered to have conversations with older family members about breastfeeding decisions, especially when living in extended family households where the older family members may be perceived as the head of the household.	Yes
Reflective motivation	Lack of self‐confidence to talk about or challenge older family members beliefs and decisions about infant feeding. Parents need to believe they are capable of initiating and holding conversations about infant feeding, and that they will be well received. Also believe that their role in this decision is important.	Yes

### 3.5 Steps 5–8: Intervention Functions; Policy Categories; BCTs; Mode of Delivery

Of the possible nine intervention functions included in the Behaviour Change Wheel, all were identified by the research team as potentially useful for addressing one or more of the behavioural targets based on the COM‐B analysis. The following five intervention functions were taken forward: education; persuasion; modelling; enablement; environmental restructuring. The intervention functions, ‘restriction’, ‘coercion’ and ‘incentivisation’ were not deemed appropriate for the interventions aims and ethos. Further, ‘training’ was considered outside of the scope of the intervention, which, as guided by the community peer group champions, was to be focussed on the development of resources for the communities which could be implemented in both family and health professional settings. The policy categories of ‘communication/marketing’ and ‘service provision’ were identified as being the most appropriate. Nine behaviour change techniques (BCTs) were selected. Table 4 lists these BCTS and illustrates how they map with the corresponding COM‐B and TDF domains (a framework that expands the elements of the COM‐B to offer a more detailed understanding of behaviour), intervention functions and policy categories.

**Table 4:** Identifying intervention options and behaviour change techniques.

Target behaviour: Parents to talk to family members about breastfeeding to establish breastfeeding support					
COM‐B components	TDF domains	Possible intervention functions	Policy category	Chosen BCTs	
Physical capability	N/A	N/A	Communication/marketing Service provision	No BCT chosen	
Psychological capability	Knowledge Cognitive and interpersonal skills	Education Training		Demonstration of behaviour [6.1]	*Animation: Scene* Father of baby holding conversations with family member about how breastfeeding can be supported *Script* Mother‐in‐law to daughter: Zahra please could you prepare dinner tonight. Don't worry I can feed Hamzah. Husband: It's OK Mum, I'll help with dinner. It's important that Hamzah is just fed by Zahra for now. Would you like a cuddle with Hamzah
Physical opportunity	N/A	N/A		No BCT chosen	
Social opportunity	Social influences	Restriction Environmental restructuring Enablement Modelling		Social support unspecified [3.1] Prompt/cue [7.1]	*Leaflet:* Support: Research shows that support from family and friends is important for breastfeeding, especially during the first few weeks. Talk to your family about how you would like to feed your baby and how they can help. You might like to read this leaflet together so they can learn how good breastfeeding is for your baby Baby groups are a good way of meeting other parents who can provide valuable support.
Reflective motivation	Social/Professional role and identity Beliefs about capabilities Beliefs about consequences	Education Persuasion Modelling Enablement		No BCT chosen	
Automatic motivation	Emotion	Persuasion Incentivisation Coercion Modelling Enablement		No BCT chosen	

Target behaviour: (Bangladeshi community) mothers to feed their baby colostrum (first breast milk)					
COM‐B components	TDF domains	Possible intervention functions	Policy category	Chosen BCTs	
Physical capability	N/A	N/A	Communication/marketing Service provision	N/A	
Psychological capability	Knowledge Cognitive and interpersonal skills Memory, attention and decision making	Education Training Environmental restructuring Enablement		Information about health consequences [5.1] Framing/reframing [13.2]	*Leaflet:* Sometimes people worry that it is not good for their baby. In fact, it is packed full of nutrients, antibodies, and energy and is everything your baby needs. Your breast milk in the first few days is a golden colour. Sometimes we call this colostrum.
Physical opportunity	N/A	N/A		N/A	
Social opportunity	Social influences	Restriction Environmental restructuring Enablement Modelling		No BCT chosen	
Reflective motivation	Beliefs about consequences	Education Persuasion Modelling		Information about health consequences [5.1] Credible source [9.1]	*Animation:* *Scene* Health professional with mother in hospital after birth *Script* Mum: ‘I was told my milk is yellow and old at the moment so do I have to wait to feed?’ Health Professional: ‘No, not at all. The first milk you produce is called colostrum. It's exactly what your baby needs at the moment. It is packed full of nutrients and antibodies to help protect baby from illness and is very gentle on his tummy.
Automatic motivation	Emotion	Persuasion Incentivisation Coercion Modelling Enablement		No BCT chosen	

Target behaviour: Parents to avoid giving their baby honey before the age of 1 year					
COM‐B components	TDF domains	Possible intervention functions	Policy category	Chosen BCTs	
Physical capability	N/A	N/A	Communication/marketing Service provision	N/A	
Psychological capability	Knowledge Memory, attention and decision making	Education Training Environmental restructuring Enablement		Information about health consequences [5.1]	*Leaflet:* Honey can cause an illness which is serious for young babies, so avoid giving honey until your baby is 1 year old.
Physical opportunity	N/A	N/A		N/A	
Social opportunity	Social influences	Restriction Environmental restructuring Enablement Modelling		No BCT chosen	
Reflective motivation	Beliefs about consequences	Education Persuasion Modelling		No BCT chosen	
Automatic motivation	Emotion	Persuasion Incentivisation Coercion Modelling Enablement		No BCT chosen	

Target behaviour*:* Parents to avoid giving their baby any taste of food or drink, aside from breast or infant formula milk, until they are at least 6 months old, and to also decline offers of this from other family members					
COM‐B components	TDF domains	Possible intervention functions	Policy category	Chosen BCTs	
Physical capability	N/A	N/A	Communication/marketing Service provision	N/A	
Psychological capability	Knowledge Memory, attention and decision making	Education Training Environmental restructuring Enablement		Information about health consequences [5.1] Prompt/cue [7.1]	*Leaflet:* Even tiny tastes of food can upset your baby's digestive system. Your baby only needs breast milk (or first infant formula) until around 6 months. Talk to your family about how you would like to feed your baby and how they can help. You might like to read this leaflet together so they can learn how good breastfeeding is for your baby
Physical opportunity	Environmental context & resources	Training Restriction Environmental restructuring Enablement		No BCT chosen	
Social opportunity	Social influences	Restriction Environmental restructuring Enablement Modelling		Demonstration of behaviour [6.1] Prompt/cue [7.1]	*Animation: Scene* Elder family member (grandma) brings over cake and offers a little bit to baby *Script* Mother: Oh no, he can't have food yet Fatima Grandma: But, just a tiny taste won't do any harm Husband: Things are a bit different these days, research shows that even tiny tastes can upset Hamzah's tummy. We'd like to wait until he is 6 months before he has anything but his milk Talk to your family about how you would like to feed your baby and how they can help. You might like to read this leaflet together so they can learn how good breastfeeding is for your baby
Reflective motivation	Beliefs about consequences	Education Persuasion Modelling		Credible source [9.1] Information about health consequences [5.1]	*Leaflet:* My health visitor told me even licking or tasting food could upset baby's tummy, so we waited until she was able to sit up before we gave anything other than milk
Automatic motivation	Reinforcement	Training Incentivisation Coercion Modelling Enablement		No BCT chose	

Target behaviour: Parents to avoid or delay the use of formula milk in place of breast milk					
COM‐B components	TDF domains	Possible intervention functions	Policy category	Chosen BCTs	
Physical capability	N/A	N/A	Communication/marketing Service provision	N/A	
Psychological capability	Knowledge Memory, attention and decision making	Education Training Environmental restructuring Enablement		Social support (practical) [3.2]	*Leaflet:* To help feel confident that your baby is growing well, you can visit a baby clinic to check your baby's weight, or speak to a health professional
Physical opportunity	Environmental context & resources	Training Restriction Environmental restructuring Enablement		No BCTs chosen	
Social opportunity	Social influences	Restriction Environmental restructuring Enablement Modelling		Information about Social and environmental consequences [5.3]	*Leaflet:* Breastfeeding anywhere: It is your legal right to breastfeed anywhere you want to, but lots of places also advertise that they are breastfeeding friendly.
Reflective motivation	Beliefs about consequences	Education Persuasion Modelling		Framing/reframing [13.2] Credible source [9.1] Information about Health consequences [5.1]	*Leaflet:* Baby's size: Every baby is different, and they come in all shapes and sizes, just like adults. Bigger is not always better. *Animation: Scene* Mum and baby at a clinic with health professional. Health professional showing weight graph and reassuring mum that baby is healthy *Script* Mum: I'm worried because Hamzah isn't as big as some of the other babies I know, is he putting on enough weight? My mother‐in‐law has said my milk isn't good milk? Health visitor: Every baby is different, just like we all are. Hamzah is growing well. As long as he keeps growing along this line then he is putting on a healthy amount of weight. Your milk is perfect for your baby and contains everything he needs
Automatic motivation	reinforcement	Training Incentivisation Coercion Modelling Enablement		Information about emotional consequences [5.6]	*Leaflet:* Having lots of close contact with you helps baby to feel safe in their new world

Initial implementation ideas were generated for each of the possible BCT's before the final intervention development workshop. During the workshop these ideas were expanded, changed or developed, specifically in relation to adjusting the language to make it appropriate and accessible for the community, along with generation of other ideas. Several considerations were discussed as important to the community peer group champions and their communities and were incorporated into the operationalisation of the BCT content in the intervention design. These were; imagery that was simple and represented a variety of cultures; abstract designs favoured over images of real breastfeeding; clearly representing the credibility of the resource; simple messages and simple language to facilitate understanding, accessibility and translation into other languages. See ‘Supporting Information: BCT content included in the LIFT intervention toolkit with detail about how they were operationalised’, for detailed information about how the intervention content maps to the BCTs used.

#### 3.5.1 Mode of Delivery

Two modes of delivery, ‘distance’ and ‘population level’ were chosen as the most appropriate for the intervention. This included print media, in the form of both posters and a ‘foldilocks’ leaflet, and digital media, in the form of an animation. Print media was viewed by all as the preferred modes of delivery. Leaflets and poster were considered in particular to be:

∘ Acceptable; viewed as the most appropriate and liked form of content to use when prompting conversations, particularly with the older generation, and noted the importance of having ‘material’ to back up their point.
∘ Practical; can be distributed in many community locations with ease, information is provided in one place, can act as a prompt resource and can be transported discreetly.
∘ Effective; the majority of the content included intentionally simple messages.
∘ Affordable; low cost materials, particularly as the intervention was to be developed in three languages.
∘ Side‐effect: Folding leaflet specifically designed to keep content hidden and reveal when this felt appropriate to avoid drawing attention to the issue before deemed appropriate by the user.
∘ Equity: Viewed as the best way to reach the older generation in their community, and easiest way to reach more of the community because it's easily disseminated. Digital or online delivery was not deemed appropriate for many within this community.

The use of apps and other interactive digital media (e.g., websites) was not considered to be the most practical way of reaching the community. Further, digital resources were not considered suitable for occasions when parents wanted to use them to prompt discussion with older generations. An animation was however considered a useful resource for use in health professional contexts, such as within workshops delivered by healthcare professionals with expectant or new parents.

### 3.6 The LIFT Intervention Toolkit

**Figure 3:**
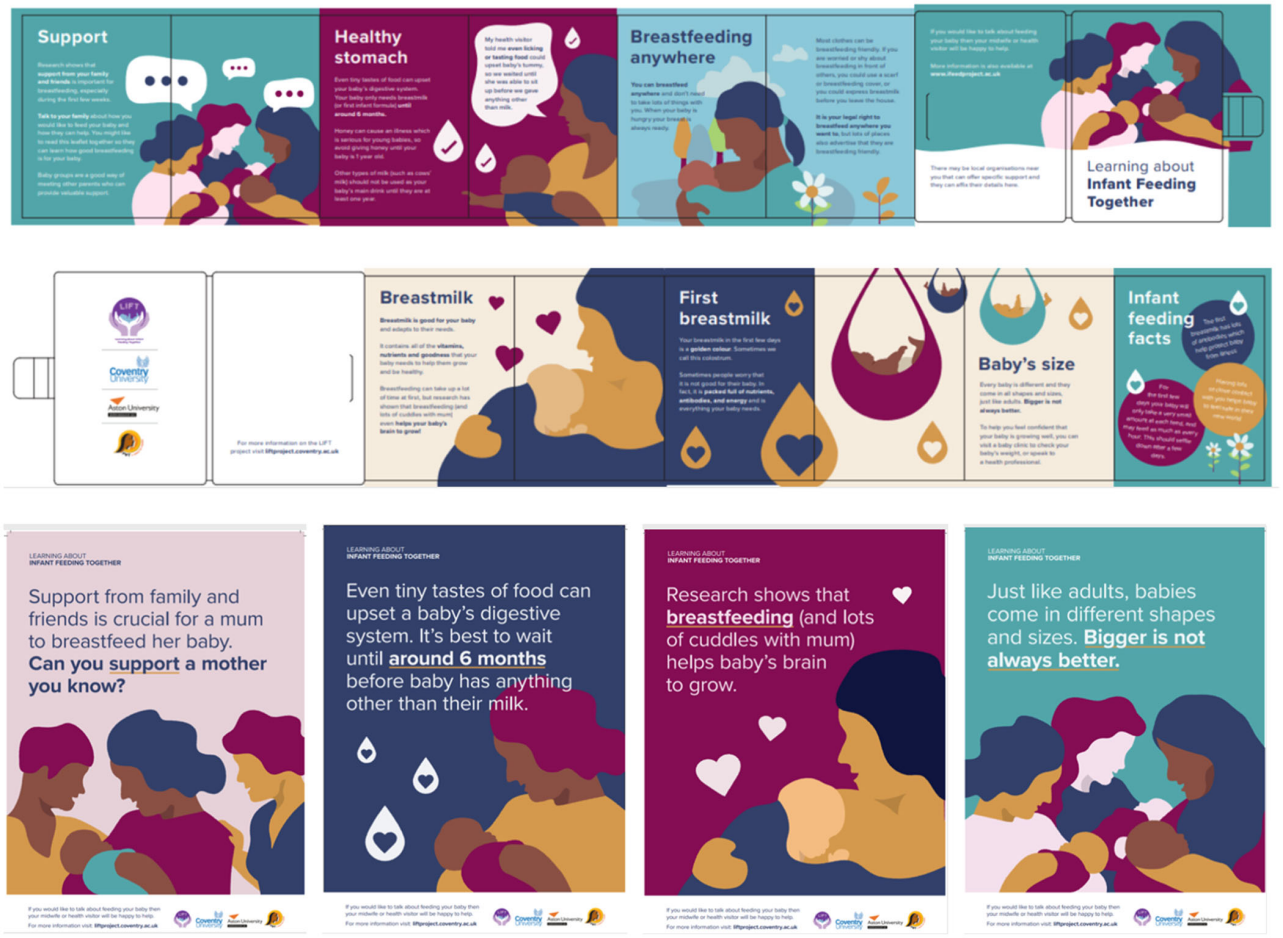
Images of the LIFT ‘foldilocks’ leaflet and the four posters co‐produced to support breastfeeding with Bangladeshi and Pakistani parents.

The LIFT intervention co‐developed and designed with the Pakistani and Bangladeshi community, consisted of a ‘toolkit’ of materials, including a leaflet, four posters, and a 3‐min animation. The leaflet and posters were produced in English, Urdu and Bengali and the animation was audio recorded in English and Urdu. Due to difficulties sourcing support to accurately record the animation in Bengali, a translation in this language could not be produced. See Figure 3 for images of the finalised intervention materials. The animation can be found online https://tinyurl.com/liftanimation-english).

### 3.7 Guidance on Toolkit Implementation

The toolkit materials are primarily designed to be used during the antenatal period, to prompt discussion about the infant feeding behaviours highlighted as pertinent to this community with healthcare professional and family members (including expectant fathers and elder relatives). The leaflets are designed for use in antenatal settings with health professionals but are easily transportable so they can be taken home and used to prompt and support further conversations with family members or the wider community. The posters are designed to be displayed in community settings, such as community centres or GP surgeries and provide short snappy messages to inform people in the community about optimal infant feeding practices and support a change in social norms. The animation was developed primarily for healthcare professionals and organisations within the community to use as a resource during antenatal classes or other relevant health promotion activities.

## 4 Discussion

This paper describes the co‐development of the LIFT intervention toolkit, a novel theory‐ and evidence‐based intervention designed to increase breastfeeding initiation and duration amongst UK Bangladeshi and Pakistani communities. Development was guided by the REPLACE approach (Barrett and Brown 2015) and involved close participatory working with community members to help ensure community relevance and acceptability. As guidance within the REPLACE manual on intervention development was limited, the project team supplemented this stage with more detailed guidance published on the Behaviour Change Wheel (Michie et al. 2014). Fidelity to the REPLACE approach was also maintained, leading to a more robust application of both frameworks simultaneously. The key behaviours identified as important and targeted by the intervention are: gaining support from family to breastfeed; feeding colostrum to baby; avoiding formula milk in place of breast milk; avoiding tastes of food or drinks other than breast milk or infant formula until 6 months; and avoiding honey until 1 year. The intervention addressed these behaviours with a multimedia toolkit that includes a leaflet, four posters and an animation. These materials incorporate BCTs that align with relevant aspects of psychological capability, social opportunity and reflective and automatic motivation identified in formative research and co‐production. As well as the target behaviours identified through the process outlined, the LIFT team along with the with the community peer group champions, discussed and included extra content that related to the UNICEF Breastfeeding Friendly Initiative standards aligned to the development of close and loving relationships between parents and their infants (UNICEF n.d.). The inclusion of these standards in services is considered ‘gold standard’ and thus vital to materials that are developed as supplementary to standard maternity care.

Maternal and infant health is a global priority and high on the UK public health agenda. Previous research has shown that South Asian populations experience context and culturally‐ specific barriers to health behaviours, emphasising the significant need for culturally tailored interventions (Lakhanpaul et al. 2020; Noor and Rousham 2008). Limited research on the development of such interventions has been conducted, and research that exists is missing detail of the intervention content and has not been developed using recognised frameworks that guide theory‐ and evidence‐based approaches (Ingram et al. 2003; Ingram and Johnson 2004). This research used the REPLACE approach (Barrett and Brown 2015) and the Behaviour Change Wheel (Michie et al. 2014) to guide development. Community engagement and formative research with the target population were central to this work. This publication provides a full account of the development process and of intervention content to aid transparency and replicability. As outlined by the MRC guidance on developing complex interventions (Skivington et al. 2021), the use of high‐quality formative research and theory‐based methodologies, coupled with clarity in reporting intervention development, has the potential to enable higher quality testing of casual pathways that will accelerate the identification of effective BCTs and support the growth of evidence‐based practice (Michie and Abraham 2004). The LIFT intervention addresses a significant gap in the application of such approaches to intervention development in infant feeding and further builds on existing formative research in this field.

The positive and successful experience of co‐developing the LIFT intervention with the community was a result of close collaboration and partnership with FWT, a community‐based organisation who work with women from South Asian communities to promote health and wellbeing. A significant amount of time and care went in to establishing positive and trusting relationship between the community and the research team which started during the formative research phase (reported elsewhere; Kwah et al. 2025). Other research, addressing infant feeding behaviour (6 months to 2 years) with the same community, and through application of a similar participatory learning and action approach (Manikam et al. 2022), demonstrates how co‐development results in resources that are viewed by the target population as appropriate and trustworthy, and subsequently championed by the community. In the present study, whilst there was a strong ethos of co‐development, and the community peer group champions were heavily involved at all stages, co‐working did present some challenges. The Behaviour Change Wheel is a complex framework, and whilst the research team endeavoured to simplify language and adapt resources to meet the needs of the group, this was more successful at some points than others. Conducting the COM‐B analysis presented the most challenge in this respect. Where peer group champions struggled with any one workshop activity, the research team took the approach of gathering as much in‐depth, relevant information as possible, and then using this to complete the activity afterwards. In such cases, this work was always presented back to the group and opportunities provided to the peer group champions to make changes or additions to ensure that their voices continued to drive decisions. One further consideration throughout this research (and highlighted in the formative research paper, see Kwah et al. 2025) was the need to remain curious and respectful to the community's beliefs and views about cultural practices that have been practiced for centuries and the meaning these hold. The REPLACE framework was a useful guide in this process, particularly the process of assessing the community's readiness to change and involving FWT and researchers belonging to the community was also vital. This research offers a demonstration of bridging the gap between the application of a rigorous theory‐based behaviour change framework with being responsive and flexible to the needs of the community peer group champions to ensure the process remains engaging and accessible to facilitate truly inclusive research design participation.

### 4.1 Limitations

Despite addressing six specific behaviours identified as interdependent with breastfeeding, there was potential to go further in terms of addressing more behaviours, or in incorporating greater numbers of or doses of BCTs. However, a significant need vocalised by the community was that the intervention should include simple resources for the purpose of starting conversations with the family about infant feeding. When making this argument they referred to existing infant feeding interventions perceived to be inaccessible and too complicated for their family members. Also, as in the case of pre‐lacteal feeds, there was recognition of the need not to go too far too fast but instead to aim for gradual change over time in line with community readiness. Though there was opportunity to include much more content, co‐development, cultural readiness/sensitivity, and the needs of the community were prioritised. The ambition is to build on this work, developing additional packages of content as and when appropriate.

A limitation in application of the methodology relates to the diversity of peer group champions. The intention was to recruit both female and male community peer group champions from across different generations, including those recognised as influential figures in their community. The final six community group peer champions were however all mothers from the community. The lack of gender diversity was related to discomfort in discussing the topic of breastfeeding with both men and women present. Further work with other members of the Pakistani and Bangladeshi community is warranted, with particular focus on involving influential figures within the community (e.g. elders, clergy, healers) to ensure full acceptability of the intervention materials. Any future work in this area should also aim for a more representative group; with workshops split by gender or other key characteristics if necessary to ensure all members feel able to express their thoughts freely. A final reflection is that individuals who volunteered as community peer group champions may have been more positive about breastfeeding and more ‘ready for change’ than counterparts in their communities, thus biasing the direction of the intervention. This issue is not however unique to this project but instead a challenge for all research involving public participation. Nonetheless, researchers must aim, as far as possible, to identity and remove barriers to involvement so that everyone, including those who have negative views towards the targeted behaviour, feels able to participate. In the present study, whilst diversity amongst peer group champions was limited, views from the wider community were represented in the formative research on which intervention content was based.

### 4.2 Implications

LIFT has been designed to be implemented in the antenatal stages to complement health professional support, though on‐going use postnatally could also be beneficial as conversations about infant feeding continue and change as the baby grows. The toolkit of resources aims to support conversations about breastfeeding between health professionals and parents, and to facilitate this also between parents and wider family members. Printed materials have been included in the toolkit as these were identified as most appropriate for use in the context of conversations about infant feeding with family members. This mode of delivery has been outlined as important to accessibility in other research, particularly when offered in other languages such as Bengali or Urdu (Ingram et al. 2003).

The next phase of planned research is to conduct a feasibility trial to assess the usability and acceptability of the materials as well as to determine whether and how a full trial to assess intervention efficacy is possible. Ahead of this, further work with grandparents, fathers, other influential figures in the community and health professionals, to understand their perspectives on what would support change, is warranted. If deemed effective, and acceptable by health professionals and the community, LIFT has the potential to be implemented at scale. Changes to infant feeding practices would directly impact infant health. A future full trial of the LIFT intervention would provide much needed primary research to establish a body of research that might begin to synthesise and make judgments about the effectiveness of BCTs on infant feeding behaviour for this population. Furthermore, the detailed and transparent reporting of the methodological approach responds to the call for a paradigm shift recommended by experts working in the field of behavioural science (Michie et al. 2018).

## 5 Conclusion

Socio‐cultural level factors are influencing infant feeding behaviours in the UK Pakistani and Bangladeshi communities and tailored interventions are warranted. The LIFT intervention was co‐developed by a team of researchers, members of a specialised 3rd sector organisation and the community. The co‐development followed principles of theory‐based frameworks alongside the community and was based on high quality formative research to elucidate the specific socio‐cultural beliefs of the target population. The process of co‐design is transparently reported here, in a bid to add to a growing body of behaviour change research and offers novel insights into developing behaviour change interventions for addressing infant feeding in this population. Further, this research provides a demonstration of successfully co‐developing a culturally appropriate intervention for infant feeding, which offers materials that can be used to complement face‐to‐face infant feeding support.

## Author Contributions

N.B., K.C., K.B., and J.B. conceptualised the study. All conducted the research, including the facilitating of intervention development workshops data collection. N.B., K.K., and M.S. analysed the data and all contributed to its interpretation. K.K. wrote the paper. K.B. provided feedback on the first draft. All read, provided feedback and approved final manuscript.

## Conflicts of Interest

The authors declare no conflicts of interest.

## Supporting information

Supporting information.

Supporting information.

Supporting information.

## Data Availability

The authors have nothing to report.
