# Supplementary material for: Application of the Behaviour Change Wheel to Optimise Infant Feeding in Bangladeshi and Pakistani Communities in the UK: Co‐Development of the Learning About Infant Feeding Together (LIFT) Intervention
Source: Matern Child Nutr. 2025 Apr 24;21(3):e70019. doi: 10.1111/mcn.70019 (PMC12150127; doi:10.1111/mcn.70019)
Supplement: Supplementary file 1 — Supporting information. [file MCN-21-e70019-s003.pdf]

Supporting Information: BCT content included in the LIFT intervention toolkit with detail about how they were operationalised

Target behaviour	Mode of delivery	Chosen BCT	Intervention content
Parents to talk to family members about breastfeeding to establish breastfeeding support	Leaflet	Social support unspecified [3.1]<br><br>Prompt/ cue [7.1]<br><br>Social Support (unspecified) [3.1]	Support: Research shows that support from family and friends is important for breastfeeding, especially during the first few weeks. Talk to your family about how you would like to feed your baby and how they can help. You might like to read this leaflet together so they can learn how good breastfeeding is for your baby<br><br>Baby groups are a good way of meeting other parents who can provide valuable support.
	Animation	Demonstration of behaviour [6.1]	*Animation Scene*<br>Father of baby holding conversations with family member about how breastfeeding can be supported<br><br>*Script*<br>Mother in law to daughter: Zahra please could you prepare dinner tonight. Don't worry I can feed Hamzah.<br>Husband: It's OK Mum, I'll help with dinner. It's important that Hamzah is just fed by Zahra for now. Would you like a cuddle with Hamzah
(Bangledeshi community) mothers to feed their baby colostrum (first breast milk)	Leaflet	Framing / reframing [13.2]	Your breastmilk in the first few days is a golden colour. Sometimes we call this colostrum.

		Information about health consequences [5.1]	Sometimes people worry that it is not good for their baby. In fact, it is packed full of nutrients, antibodies, and energy and is everything your baby needs.
	Animation	Framing / reframing [13.2]<br><br>Information about health consequences [5.1]<br><br>Credible source [9.1]	*Scene*<br>Health professional with mother in hospital after birth<br><br>*Script*<br>Mum: "I was told my milk is yellow and old at the moment so do I have to wait to feed?"<br>Health Professional: "No, not at all. The first milk you produce is called colostrum. It's exactly what your baby needs at the moment. It is packed full of nutrients and antibodies to help protect baby from illness, and is very gentle on his tummy.
Parents to avoid giving their baby any taste of food or drink, aside from breast or infant formula milk, until they are at least 6 months old, and to also decline offers of this from other family members	Leaflet	Information about health consequences [5.1]	Even tiny tastes of food can upset your baby's digestive system. Your baby only needs breastmilk (or first infant formula) until around 6 months.<br><br>The first breastmilk has lots of antibodies which help protect baby from illness
		Credible source [9.1]<br>Information about health consequences [5.1]	My health visitor told me even licking or tasting food could upset baby's tummy, so we waited until she was

			able to sit up before we gave anything other than milk
		Prompt/cue [7.1]	Talk to your family about how you would like to feed your baby and how they can help. You might like to read this leaflet together so they can learn how good breastfeeding is for your baby
	Animation	Demonstration of behaviour [6.1]	*Animation Scene*<br>Elder family member (grandma) brings over cake and offers a little bit to baby<br><br>*Script*<br>Mother: Oh no, he can't have food yet Fatima<br>Grandma: But, just a tiny taste won't do any harm<br>Husband: Things are a bit different these days, research shows that even tiny tastes can upset Hamzah's tummy. We'd like to wait until he is 6 months before he has anything but his milk
Parents to avoid giving their baby honey before the age of 1 year	Leaflet	Information about health consequences [5.1]	Honey can cause an illness which is serious for young babies, so avoid giving honey until your baby is 1 year old.
Parents to avoid or delay the use of formula milk in place of breast milk	Leaflet	Information about health consequences [5.1]	Breastmilk: Breastmilk is good for your baby and adapts to their needs. It contains all of the vitamins, nutrients and goodness that your baby needs to help them grow and be healthy

		Information about emotional consequences [5.6]<br><br>Framing/reframing [13.2]<br><br>Social support (practical) [3.2]<br><br>Information about Social and environmental consequences [5.3]	Breastfeeding can take up a lot of time at first, but research shows that breastfeeding) and lots of cuddles with mum) even helps your baby's brain to grow!<br><br>Having lots of close contact with you helps baby to feel safe in their new world<br><br>Baby's size: Every baby is different and they come in all shapes and sizes, just like adults. Bigger is not always better.<br><br>To help feel confident that your baby is growing well, you can visit a baby clinic to check your baby's weight, or speak to a health professional<br><br>Breastfeeding anywhere: It is your legal right to breastfeed anywhere you want to, but lots of places also advertise that they are breastfeeding friendly.
	Animation	Credible source [9.1]<br><br>Information about Health consequences [5.1]	*Animation Scene*<br>Mum and baby at a clinic with health professional. Health professional showing weight graph and reassuring mum that baby is healthy<br><br>*Script*<br>Mum: I'm worried because Hamzah isn't as big as some of the other babies

			I know, is he putting on enough weight? My mother in law has said my milk isn't good milk?<br>Health visitor: Every baby is different, just like we all are. Hamzah is growing well. As long as he keeps growing along this line then he is putting on a healthy amount of weight. Your milk is perfect for your baby and contains everything he needs
