# Supplementary material for: Application of the Behaviour Change Wheel to Optimise Infant Feeding in Bangladeshi and Pakistani Communities in the UK: Co‐Development of the Learning About Infant Feeding Together (LIFT) Intervention
Source: Matern Child Nutr. 2025 Apr 24;21(3):e70019. doi: 10.1111/mcn.70019 (PMC12150127; doi:10.1111/mcn.70019)
Supplement: Supplementary file 2 — Supporting information. [file MCN-21-e70019-s001.pdf]

**Supporting Information: Identifying what needs to change using the COM B model**

| **Target behaviour:** Parents to talk to family members about breastfeeding to establish breastfeeding support | | |
|---|---|---|
| **COM-B component** | **Evidence related to component & if relevant what needs to happen for the target behaviour to occur?** | **Is there a need for change?** |
| Physical capability | There is no evidence that holding the verbal and language skills to hold a conversation with family members is a barrier to establishing support to breastfeeding, as in most cases there are no communication or language barriers. | No |
| Psychological capability | Being able to explain their feeding choices and the knowledge based used to make this decision, to other family members, respectfully in the relationship they hold was highlighted as a barrier to establishing breastfeeding support.<br><br>Being knowledgeable about the benefits of breastfeeding and confident about the signs of healthy infant growth is faciliatory to these conversations. Furthermore, being able to express thoughts and feeling about infant feeding decisions, and any support needed, to elder family members is a facilitator to establishing breastfeeding support. | Yes |
| Physical opportunity | No evidence of physical opportunity being a barrier to establishing breastfeeding support, as parents are able to be physically present in a space with older family members. | No |
| Social opportunity | Mother-in-laws have a significant role in infant feeding decisions, especially when living in extended families and some have engrained negative thoughts/ feeling about breastfeeding and this can act as a barrier to starting conversations.<br><br>Parents need to feel like they can ask family members for support and family members need to open to conversations about infant feeding benefits, decision and the support needed, and open to hearing new knowledge-based perspectives that might not align to their own. | Yes |
| Automatic motivation | Parents don’t want to cause offense or be disrespectful of their older family members beliefs.<br><br>Parents need to be empowered to have conversations with older family members about breastfeeding decisions, especially when living in extended family households where the older family members may be perceived as the head of the household. | Yes |
| Reflective motivation | Lack of self-confidence or competence to talk about or ‘go against’ older family members beliefs and decisions about infant feeding.<br><br>Parents need to believe they are capable of initiating and holding conversations about infant feeding, and that they will be well received. Also believe that their role in this decision is important. | Yes |

| **Target behaviour:** (Bangledeshi) mothers to feed their baby colostrum (first breast milk) | | |
|---|---|---|
| **COM-B component** | **Evidence related to component & if relevant what needs to happen for the target behaviour to occur?** | **Is there a need for change?** |
| Physical capability | No evidence that physical capability is a barrier to feeding colostrum. | No |
| Psychological capability | Some mothers have beliefs about colostrum being old or ‘dirty before birth of baby.<br><br>Mothers need to have knowledge that colostrum is nutritionally rich and tailored for their baby's needs. Need to have knowledge that colostrum is not old milk. Interpersonal skills to relay this health knowledge to others (older family members). | Yes |
| Physical opportunity | No evidence that the physical opportunity is a barrier to feeding colostrum. Birthing setting to have health professionals present who can assist with immediate skin-to skin and latching baby to breastfeed/ not being in the presence of people telling them to discard colostrum is a facilitator to feeding colostrum. | No |
| Social opportunity | Mothers who don’t feed colostrum due to belief that it is old or ‘dirty’ are likely to have had heard this information from older family members. Older family member, especially mother-in law heavily involved with decisions around infant feeding.<br><br>Mothers need support from others (family members) to feed colostrum and not being told / persuaded to discard it in favour of infant formula milk would facilitate the feeding of colostrum. | Yes |
| Automatic motivation | Not feel worried about feeding baby colostrum or worried about what others might think or say about it. | Yes |
| Reflective motivation | Some people have cultural beliefs that colostrum is not good for their baby as this acts as a barrier to feeding colostrum.<br><br>Mothers need to believe that colostrum is good milk for their baby and that they are making the best choice for them, accepting that might be different to advice being passed down from older family members would acts a s a facilitator to feeding colostrum. | Yes |

| ***T*arget behaviour:** Parents to avoid giving their baby honey before the age of 1 year | | |
|---|---|---|
| **COM-B component** | **Evidence related to component & if relevant what needs to happen for the target behaviour to occur?** | **Is there a need for change?** |
| Physical capability | No evidence to show that physical capability is a barrier to avoiding giving honey to baby under 1 year. | No |
| Psychological capability | Giving honey to an infant (under 1 years old) is not considered harmful.<br><br>Parents need to have the knowledge about why giving honey is a risk to an infant's health and also need to be able to explain to others the reasoning behind changing this practice without causing offence. | Yes |
| Physical opportunity | Depending on where the baby is birthed, physical opportunity to perform practice may be hampered by hospital rules around visitors | No |
| Social opportunity | Parents may feel obliged due to family / cultural traditions or religious teachings. Elders may take offence if not asked to perform ritual. Suggestions to change or replace the ritual with an alternative may be met with resistance if it is what has been done for generations without prior evidence of harm. May also be seen as a religious requirement.<br><br>Parents need to say no (specifically about honey) to a social pressure against a social / cultural norm in favour of trying to get social support about following health guidance for their baby. | Yes |
| Automatic motivation | Belief that good qualities pass from elder to baby; emotions that come with traditional ritual around giving birth and passing on down generations; gives value to older generation and marks new life.<br><br>Parents need to feel positively about not allowing honey to be given to their infant, that giving honey is not the only way to reinforce passing on good family qualities. | Yes |
| Reflective motivation | Behaviour of giving honey is not seen as harmful, because it is just a small/ tiny taste. Has been done for generations without prior evidence of harm.<br><br>Parents need to believe that making the choice to not give honey to an infant is in the best interest of the infant's health and having the confidence to stick with this decision. | Yes |

| **Target behaviour:** Parents to avoid giving their baby any taste of food or drink, aside from breast or infant formula milk, until they are at least 6 months old, and to also decline offers of this from other family members |
|---|

| COM-B component | Evidence related to component & if relevant what needs to happen for the target behaviour to occur? | Is there a need for change? |
|---|---|---|
| Physical capability | No evidence that physical capability is a barrier to avoiding tastes of food as in most cases the parents have the verbal and language skills to decline offer of food/ drink ‘tastes. | No |
| Psychological capability | While parents are able to not offer tastes, they may face barriers in terms of their families' offering foods. Sometimes the way the foods are described indicated that actually baby was given some foods, but it wasn’t perceived as such because it was ‘just a taste’.<br><br>Understand that any taste of food or drink that is not breast or infant formula milk is considered giving solids before 6 months of age. Have a good understanding of the negative effects of food/ drink under the age of 6 months. Be able to confidently relay this information to other family members in a respectful way, especially older family members. | Yes |
| Physical opportunity | It is known that sometimes family members may give baby tastes without the consent of the parent/ in the absence of the parent.<br><br>Parents need to be physically present at the time the baby is offered ‘tastes’ to refuse them. | Yes |
| Social opportunity | Offering ‘tastes’ is a cultural norm. Parents are not always seeing other parents who are like them also declining to offer their infant tastes of food and drinks before 6 months.<br><br>Parents need to have other people around them who are also saying no to tastes of food for their baby under 6 months old. Support from other family members to respect parents' decisions to not give food or tastes to their infant. | Yes |
| Automatic motivation | When baby is poorly parents may automatically react by giving them honey/ water with aniseed etc.<br><br>Parents need to be able to challenge the habit or reinforcement from others about giving baby tastes. | Yes |
| Reflective motivation | Cultural beliefs ingrained through generations may influence self-conscious intention to give tastes as they are seen as having benefits to baby's health.<br><br>Parents and other family members need to hold a belief that the consequences of offering tastes are harmful to the infant’s health. | Yes |

| |
|---|
| **Target behaviour:** Parents to avoid or delay the use of formula milk in place of breast milk |

| **COM-B component** | **Evidence related to component & if relevant what needs to happen for the target behaviour to occur?** | **Is there a need for change?** |
|---|---|---|
| Physical capability | No evidence that physical capability is a barrier to avoiding formula milk in place of breastfeeding. | No |
| Psychological capability | Belief that with formula you can know exactly how much milk the baby is getting, and what vitamins. Choosing formula is confusing, numbers on tubs difficult to understand.<br><br>Need the confidence to seek reassurance that baby is receiving enough milk. Need the interpersonal skills to discuss with other family members/ elders the benefits of breast milk. | Yes |
| Physical opportunity | Formula is expensive and required time to clean, sterilise etc. Family members offer to give baby formula milk to help. No time to breastfeed in extended families, because of other household responsibilities. Being in public without a cover or breastfeeding compatible clothing, needing more places to be able to feed when out of the house.<br><br>Parents need offers of support with other household responsibilities to allow time to breastfeed and either breastfeeding covers or breastfeeding spaces in public with suitable privacy. | Yes |
| Social opportunity | Older family members have a significant role in decisions about infant feeding, especially when living in extended families. Older family members often the source of comments about baby being 'weak' or milk not being good enough, and expectations around mothers' behaviour e.g. cannot breastfeed baby front of family/ in public). Husbands are not seen to have a role in breastfeeding. It is also viewed that bottle feeding allows for feeding in public, but breastfeeding does not.<br><br>Parents need support from other family members to breastfeed, that isn't offering to give a bottle. Both parents to talk and be involved in breastfeeding and able to talk to other family members about it. | Yes |
| Automatic motivation | Bottle feeding is seen as social norm in the UK, and breastfeeding is not valued as highly by some. Belief that baby 'needs to get used to bottle' and that breastfeeding babies are clingy.<br><br>Parents need to believe that breastfeeding is valuable and provides more than nutrition. | Yes |
| Reflective motivation | Belief that formula provides all the vitamins baby needs, it easy to know how much they are getting, and it fills them up. Belief that formula makes babies big and strong and that breastfeeding babies are small. Also awareness around risk of overfeeding and infection.<br><br>Parents need to believe that breastfeeding is good for their baby, and that their baby is receiving the right amount of milk / nutrition to grow and be healthy. | Yes |
