# Supplementary material for: Application of the Behaviour Change Wheel to Optimise Infant Feeding in Bangladeshi and Pakistani Communities in the UK: Co‐Development of the Learning About Infant Feeding Together (LIFT) Intervention
Source: Matern Child Nutr. 2025 Apr 24;21(3):e70019. doi: 10.1111/mcn.70019 (PMC12150127; doi:10.1111/mcn.70019)
Supplement: Supplementary file 3 — Supporting information. [file MCN-21-e70019-s002.pdf]

Supporting Information: Long list of possible intervention actions/ target behaviours generated during the first intervention development workshop

Community	Key beliefs and practices	Stage of community readiness to change	Target actions/ behaviour
Pakistani & Bangladeshi	Breastfeeding decisions and behaviour influenced by family members (e.g. mother-in-law make decision)<br><br>The baby's father doesn't have a role in infant feeding behaviour<br><br>Daughter-in-law household duties should be done before feeding the baby<br><br>Belief that formula milk is easier because mothers have household duties that make breastfeeding more difficult<br><br>'Pardah' or the need for modesty, making bottle feeding easier and breastfeeding more difficult in the compay of others	Medium<br><br>Focus on individual empowerment- change attitudes & initiate behaviour change	1. Mother to establish responsive breastfeeding<br><br>2. Mother/ parents to establish breastfeeding support from other family members before the baby is born by talking to them about breastfeeding<br><br>3. Mother and father to be to have a conversation with family members about intending to breastfeeding their baby, why they have made this choice and how they can support that<br><br>4. Professionals to talk to parents and grandparents (elders) with focus on positive messaging about breastfeeding and its importance to baby and the supportive role they can play<br><br>5. Fathers offer to help complete household duties to allow mothers to breastfeed baby<br><br>6. Younger generation grandparents (who are breastfeeding supporters) to persuade and influence older grandparents (who are not breastfeeding advocates)

Bangladeshi	Colostrum (first breast milk) is old and dirty and is discarded before the infants first feed	Medium<br><br>Focus on individual empowerment- change attitudes & initiate behaviour change	1. Mothers to feed their baby colostrum (first breast milk) when born<br><br>2. Health professionals talk to mothers about what colostrum is, what it looks like and the importance of this first breast milk (including antibodies)<br><br>2. Health professionals to discuss colostrum antenatally / at the time of birth<br><br>3. Mothers to discuss with older family members why they will be feeding their baby colostrum
Pakistani & Bangladeshi	Pre-lacteal feed or 'Ghutti' passes on important qualities from family elder to baby and may be seen as a religious teaching	Low<br><br>Focus on community empowerment – increase knowledge	1. Health professionals or community workers to talk to all family members about the potential negative health implications of honey for a baby under 1<br><br>2. Health professionals or community workers to talk to community members about the potential negative health implications of honey for a baby under 1<br><br>3. Family members to discuss other ways to pass on good qualities from elders to baby<br><br>4. Parents to say no to giving their baby honey
Pakistani & Bangladeshi	Giving 'tastes' of other foods/ drinks before 6 months of age	Medium	1. Parents chose not to give baby under 6 months any taste of food or drink, aside from breast or infant formula milk

		Focus on individual empowerment- change attitudes & initiate behaviour change	2. Parents say no to family or community members when they ask/ offer baby under 6 months food (anything other than breast or infant formula milk)<br><br>3. Organisations to run community session to involved new parents and the older generation focussed on teaching about the negative impact of food or ‘tiny tastes’ on a baby’s digestive system before 6 months of age.<br><br>4. Health professionals to talk to the older generation to explains why babies should not be offer anything besides breast or infant formula milk until around 6 months
Pakistani & Bangladeshi women	Some mother’s milk is not good enough quality and results in a smaller, less nourished baby, who needs to be given formula milk<br><br>Belief that formula milk = bigger and healthier baby (mostly from the elder generation)	Medium<br><br>Focus on individual empowerment- change attitudes & initiate behaviour change	1.Organisations/ health professionals to talk to parents and grandparents (elders) with focus on message that every baby is different, milk is different for every woman<br><br>2. Parents to avoid or delay the use of formula milk in place of breast milk<br><br>3. Health professionals to talk to mothers and other family members about the composition of breastmilk and how to spot signs of growth / nourishment

Stages of community readiness to change

Low = No community awareness / community denial/ resistance or vague community awareness

Medium = Preplanning /preparation / Initiation (medium)

High = Stabilisation / expansion / Community ownership
